# A Stepwise Framework for the Systematic Development of Lipid Nanoparticles

**DOI:** 10.3390/biom12020223

**Published:** 2022-01-27

**Authors:** João Basso, Maria Mendes, Tânia Cova, João Sousa, Alberto Pais, Ana Fortuna, Rui Vitorino, Carla Vitorino

**Affiliations:** 1 Faculty of Pharmacy, University of Coimbra, 3000-548 Coimbra, Portugal; joaobasso@ff.uc.pt (J.B.); mariamendes1093@gmail.com (M.M.); jjsousa@ff.uc.pt (J.S.); afortuna@ff.uc.pt (A.F.); 2 Coimbra Chemistry Centre, Department of Chemistry, University of Coimbra, 3004-535 Coimbra, Portugal; tfirmino@qui.uc.pt (T.C.); pais@qui.uc.pt (A.P.); 3 Coimbra Institute for Biomedical Imaging and Translational Research, University of Coimbra, 3000-548 Coimbra, Portugal; 4 iBiMED—Department of Medical Sciences, University of Aveiro, 3810-193 Aveiro, Portugal; rvitorino@ua.pt; 5 UnIC, Department of Surgery and Physiology, Faculty of Medicine, University of Porto, 4200-319 Porto, Portugal; 6 QOPNA & LAQV-REQUIMTE, Chemistry Department, University of Aveiro, 3810-193 Aveiro, Portugal

**Keywords:** drug formulation, lipid nanoparticles, multivariate analysis, NLCs, screening, SLNs

## Abstract

A properly designed nanosystem aims to deliver an optimized concentration of the active pharmaceutical ingredient (API) at the site of action, resulting in a therapeutic response with reduced adverse effects. Due to the vast availability of lipids and surfactants, producing stable lipid dispersions is a double-edged sword: on the one hand, the versatility of composition allows for a refined design and tuning of properties; on the other hand, the complexity of the materials and their physical interactions often result in laborious and time-consuming pre-formulation studies. However, how can they be tailored, and which premises are required for a “right at first time” development? Here, a stepwise framework encompassing the sequential stages of nanoparticle production for disulfiram delivery is presented. Drug in lipid solubility analysis leads to the selection of the most suitable liquid lipids. As for the solid lipid, drug partitioning studies point out the lipids with increased capacity for solubilizing and entrapping disulfiram. The microscopical evaluation of the physical compatibility between liquid and solid lipids further indicates the most promising core compositions. The impact of the outer surfactant layer on the colloidal properties of the nanosystems is evaluated recurring to machine learning algorithms, in particular, hierarchical clustering, principal component analysis, and partial least squares regression. Overall, this work represents a comprehensive systematic approach to nanoparticle formulation studies that serves as a basis for selecting the most suitable excipients that comprise solid lipid nanoparticles and nanostructured lipid carriers.

## 1. Introduction

Disulfiram (DSF) is a dithiocarbamate derivative that has long been used in the clinic to treat alcohol addiction. As an inhibitor of hepatic aldehyde dehydrogenase 1 and 2, it increases blood acetaldehyde levels upon alcohol consumption, leading to nausea, sweating, respiratory distress, hypotension, and other alcohol intoxication symptoms [1]. Recently, it has shown promising results in reducing the cell viability of different types of cancer, with a possible effect on at least nineteen different targets or pathways. Nonetheless, clinical trials have failed to support the in vitro/in vivo findings, which is most likely due to DSF’s low half-time (extensive hepatic metabolism and serum degradation) and aqueous solubility (0.2 g/L) [2]. Several anticancer drugs are also hydrophobic. Consequently, they are usually poorly absorbed, have low oral bioavailability, and cannot be administered parenterally [3]. The use of nanotechnology may circumvent this issue by providing a strategy to improve drug bioavailability in tumor tissues and ensure an appropriate therapeutic effect.

**Figure 1:**
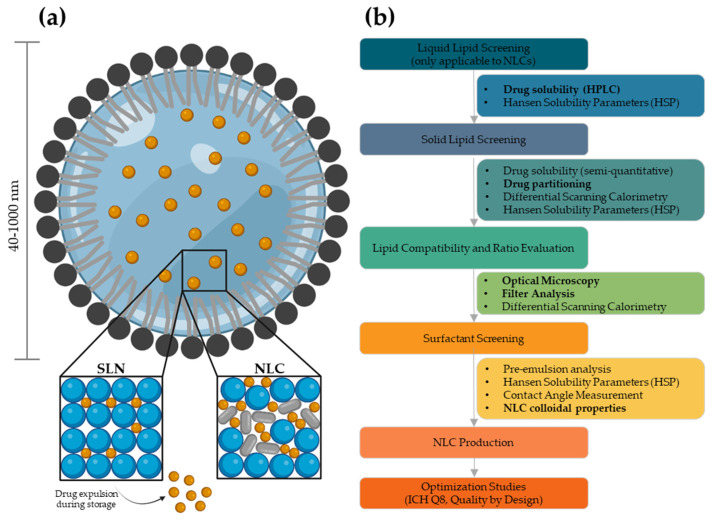
(**a**) Structure of solid lipid nanoparticles (SLNs) and nanostructured lipid carriers (NLCs), composed of a solid lipid matrix stabilized by an outer surfactant shell. The first are composed by an organized crystalline structure with identically shaped molecules, limiting drug loading, whereas the latter benefits from the introduction of a liquid lipid, creating structural imperfections and increasing drug loading and stability. (**b**) Stepwise workflow for the development of lipid nanoparticles. The experimental techniques used in this work are highlighted in bold.

Solid lipid-based nanoparticles are prominent drug delivery systems composed of a solid lipid core, either in amorphous or crystalline state, stabilized by one or more surfactants, which are usually non-ionic and/or cationic (Figure 1a). Lipid nanoparticles stem from oil-in-water emulsions in which the introduction of a solid lipid at room and body temperature leads to the formation of a solid core. Similar to polymeric systems, this solid matrix is responsible for the controlled release of active substances as well as chemical and physical protection against degradation [4].

Lipids are known to exhibit different polymorphic forms with varying stability. During preparation, part of the lipids recrystallizes in a higher energy modification (α or ß’). During storage, the reorganization of the structural crystal lattice may occur, thus evolving to a more thermodynamic stable configuration (ß). Consequently, the lipid matrix rearranges into a highly ordered system, thus promoting drug leakage [5,6]. Solid lipid nanoparticles (SLNs), the first generation of lipid nanoparticles, are made exclusively from solid lipids. Although they are considered promising drug delivery systems because they have better biocompatibility and biodegradability, there are often problems with drug expulsion and stability. In turn, a second generation of lipid nanoparticles has been developed: nanostructured lipid carriers (NLCs) benefit from the introduction of a liquid lipid that reduces the crystallinity of the lipid matrix and consequently increases drug loading [7]. Their particle size typically ranges between 150 and 300 nm, although smaller (>40 nm) and bigger (<1000 nm) carriers can be developed as needed. Note that lipid nanoparticles with larger size (>700 nm) tend to be less stable due to flocculation and creaming. On the other hand, nanoparticles with smaller size usually require higher concentrations of surfactants that may increase toxicity [8]. In addition, ultra-small carriers are effectively filtered by the kidneys, whereas bigger nanoparticles (>200 nm) are phagocytized by immune cells [9,10].

SLNs and NLCs are incredibly versatile in their composition, as they benefit from the extensive availability of materials in the cosmetic, food, and pharmaceutical industry. Researchers may hand-pick excipients with different properties: for example, fatty acids differing in chain length and/or in saturations have different melting points, serving as solid (e.g., stearic acid) or liquid (e.g., oleic acid) lipids. Similarly, glyceride esters (either mono-, di- or diacylglycerides) with variable fatty acid chains and phospholipids are also potential materials for the construction of the lipid matrix, the latter exhibiting an amphiphilic behavior and thus also acting as surfactants [11]. Yet, the regulatory status of the excipients should also be taken into consideration. Lipids (and surfactants) that hold the status of “generally recognized as safe” (GRAS) should be preferred, as they are less likely to cause acute and chronic toxicity. Nonetheless, there is flexibility in the choice of excipients: formulations for topical delivery may benefit from less strict regulations. However, these increase for oral and especially for parenteral administration [4]. In spite of this, solid lipid nanoparticles, either SLNs or NLCs, are considered safe carriers for drug delivery [12]. For intravenous administration, several medium and long-chain triglycerides, as well as fatty acids (lauric, oleic, palmitic, linoleic, and α-linoleic acids) are options [13]. In parallel, the use of phospholipids (from soybean oil and egg yolk) and polymeric surfactants such as Tween 80 (polysorbate 80), poloxamer 188, and low molecular weight polyvinyl pyrrolidone have a long-time safety established [4]. Over the last few years, several excipients have been approved by the FDA in their ‘Inactive Ingredients Database’, opening the possibility for the development of novel injectable drug nanocarriers.

There are no strict rules for selecting the best composition of NLCs, although some points should be considered: (i) acceptable solubility in liquid lipids increases drug loading and entrapment efficiency; (ii) acceptable solubility in solid lipids favors controlled release of the drug as well as protection against degradation; (iii) good compatibility between lipids promotes good uniformity and stability of the formulation; (iv) good emulsification performance of the surfactant(s) generates particles with reduced size, monodisperse, and with increased stability (Figure 1b). Further optimization studies can be conducted, as needed, to achieve ideal particle size and size distribution, a more or less controlled release (based on the liquid/solid lipid ratio), positive zeta potential values (recuring to cationic surfactants), and tumor targeting abilities (using surface-modification strategies). The use of Quality by Design strategies for pharmaceutical development is highly encouraged, with lipid content, drug loading, surfactant concentration, and process parameters as factors for optimization [14,15,16,17].

Here, a systematic stepwise strategy is set forth for the development of NLCs encapsulating DSF as an anticancer model drug. Emphasis is placed on pre-formulation studies that can serve as a basis for determining the most appropriate excipients that compose solid lipid-based nanoparticles. Such an approach should enable the finished product to consistently meet its predefined characteristics from the outset so that it is ‘right the first time’.

## 2. Materials and Methods

### 2.1. Materials

Disulfiram (CAS number 97-77-8), Kolliphor RH40, Myrj 52, oleic acid, Tween 20, and Tween 80 were purchased from Sigma-Aldrich (Saint Louis, WI, USA). Apifil, Capryol 90, Capryol PGMC, cetylpalmitate, Compritol 888 ATO, Labrafac Lipophile WL 1349, Labrafac PG, Lauroglicol 90, Geleol FPF, Geleol mono/dyglicerides NF, Geloil SC, Labrafil M 2125 CS, Labrafil 1944 CS, Labrasol, Labrasol ALF, Monosteol, Precirol Ato 5, Softisan 601, Suppocire CM, Suppocire DM, Suppocire NB, Suppocire CS2X, and Transcutol HP were kindly gifted by Gatefossé (Lyon, France). Capmul MCM and Mygliol 812 N were provided by Abitec (Columbus, OH, USA). Dynasan 116, Dynasan 118, Inwitor 900 F, Witepsol E76, and Witepsol E85 were donated by IOI Oleochemical (Hamburg, Germany). Lipoid S75 was gifted by Lipoid GmbH (Ludwigshafen, Germany). Kolliwax CA, Kolliwax CSA, Kolliwax GMS II, Kolliwax S, Kolliphor P188, Kolliphor ELP, Kolliphor HS15, and Tween 60 were provided by BASF (Ludwigshafen, Germany). Squalene and Squalane were acquired from EFP Biotek (Figueira da Foz, Portugal). Tween 40, Span 20, Span 40, Span 60, and Span 80 were gifted by SEPPIC SA (Paris, France). Water (Ω = 18.2 MΩ.cm, TOC < 1.5 µg/L) was ultrapurified (Sartorius^®^, Gottingen, Germany) and filtered through a 0.22 µm nylon filter prior to use. All the other reagents were analytical or High Performance Liquid Chromatography (HPLC) grade.

### 2.2. Methods

#### 2.2.1. Lipid Screening

##### Liquid Lipid Screening

Drug in lipid solubilities is a major requisite for the production of NLCs. For that, an excess of DSF was dispersed in screw-capped glass vials containing each liquid lipid and magnetically stirred at 25 °C for 48 h to ensure lipid saturation. Samples were centrifuged (11,740× *g*) at 25 °C for 10 min, filtered through a 0.2 µm polytetrafluoroethylene (PTFE) membrane, suitably dissolved in acetonitrile or methanol (depending on lipid miscibility with the organic solvent), and DSF was quantified by HPLC. Each determination was carried out in triplicate.

##### Solid Lipid Screening

The solid lipid was chosen based on the DSF partition between the lipid and the aqueous phases. First, DSF (5% *w*/*w*) was dispersed in a binary mixture of lipid and ultrapurified water and incubated in a temperature-controlled vortex agitator at 10 °C above the melting point of the lipid for 1 h. Following this period, the lipids were allowed to recrystallize. In order to quantify the dissolved or dispersed DSF, lipid blocks were washed with ultrapurified water and melted. A certain volume of each lipid was suitably diluted in mobile phase and sonicated for 15 min at 75 °C. Then, the resulting solution was centrifuged (11,740× *g*) at 4 °C for 5 min, filtered through a 0.2 µm PTFE membrane, and analyzed by HPLC. Each determination was performed in triplicate.

##### Lipid Compatibility

The compatibility and miscibility of the solid and liquid lipids were evaluated by optical microscopy, macroscopic analysis, and recurring to the paper filter test. Binary mixtures containing the solid and liquid lipids were heated and homogenized in screw-capped glass vials at 10 °C above the melting point of the solid lipid and homogenized. For the microscopic observation, a droplet of each mixture was transferred to a hot microscope glass slide, covered with a coverslip, and left to cool and recrystallize, whereas for the paper filter test, the cooling process occurred without the cover glass. The adsorption of lipid droplets onto the filter paper indicates a non-solid mixture of lipids and therefore lipid incompatibility. On the contrary, the absence of oil stains indicates good miscibility and solidification of the mixture. Visual inspection of the vials was performed to detect possible phase separation and mixture heterogeneity.

#### 2.2.2. Surfactant Screening

The selection of the surfactant is critical to obtain stable formulations with desired colloidal properties. NLCs were produced by the high-pressure homogenization method, as described elsewhere [17]. For that, DSF (5% *w*/*w*) was added to the molten lipid phase (10% *w*/*w* of lipid content, 3 g) containing the lipid mixture, at a 50:50 ratio, as well as the oily phase surfactant, set at 1% *w*/*w*. The mixture was added to 30 mL of the aqueous phase, containing the respective surfactant, set at 5% *w*/*w*, and emulsified for 1 min at 24,000 rpm with an Ultra-Turrax X 10/25 (Ystral GmbH, Dottingen, Germany). Then, this pre-emulsion was transferred to a pre-heated high-pressure homogenizer, Emulsiflex^®^ C3 (Avestin Inc., Ottawa, ON, Canada) and processed at 1000 bar for further 7.5 min. During the production, the temperature was kept at 10 °C above the melting point of the respective solid lipid. The dispersion was cooled and stored at 4 °C to promote matrix recrystallization and nanoparticle formation.

#### 2.2.3. Particle Size and Zeta Potential Analysis

The average particle size and polydispersity were determined by dynamic light scattering (DLS), whereas zeta potential was measured by electrophoretic light scattering, recurring to a Zetasizer Nano ZS (Malvern Instruments, Malvern, UK), with Zetasizer V7.13. The particle size, PS, was evaluated with a backward scattering angle of 173°, with the hydrodynamic diameter being calculated according to the Stokes–Einstein Equation (1),

PS = (K_B_ *T*)/(3 π η D) (1)

where K_B_ corresponds to the Boltzmann’s constant, *T* is the absolute temperature, η is the viscosity of the dispersion media, and D is the translational diffusion coefficient [18].

Zeta potential, ZP, as an indicator of the particle’s surface charge, derives from the Henry–Smoluchowski Equation (2),

*U*_E_ = (2 ε ZP f(κα))/(3 η) (2)

where *U*_E_ corresponds to the eletrophoretic mobility and ε and η correspond to the dieletric constant and viscosity of the media, respectively. f(κα) is Henry’s function and assumes a value of 1.5 [19].

Both measurements were performed after a 100-fold dilution of NLCs in ultrapurified water, at 25 °C, and analyzed in triplicate.

#### 2.2.4. HPLC Analysis

DSF was quantified by reversed-phase high pressure liquid chromatography (RP-HPLC), in a Shimadzu LC-2010HT chromatographic system, recurring to a previously optimized and validated method, per FDA and ICH guidelines [20]. Briefly, the separation of DSF was achieved using an ACE^®^ 5 C_18_ column, with 150 mm of length, 4.6 mm of internal diameter, and 5 µm of particle size (Advanced Chromatography Technologies Ltd., Reading, UK), supported by a SecurityGuard C_18_ cartridge. The analytical samples were suitably diluted in the mobile phase (70:30 (*v*/*v*) acetonitrile:water), except when otherwise indicated, with 10 µL being analyzed at a flow rate of 1 mL/min, under isocratic conditions, at 40 °C. DSF was eluted at 3.9 min and detected at 217 nm, with a limit of quantification of 0.10 µg/mL.

#### 2.2.5. Entrapment Efficiency and Drug Loading

The entrapment efficiency (EE) and drug loading (DL) were calculated indirectly through the quantification of the free drug present in the aqueous phase of the dispersions. The entrapment efficiency, i.e., the ratio between the amount of drug present in the nanoparticles and the aqueous phase, was determined according to Equation (3),

%EE = (W_total DSF_ − W_free DSF_)/W_total DSF_ × 100. (3)

The drug loading, i.e., the total amount of drug dissolved or dispersed in the lipid phase is given by Equation (4),

%DL = (W_total DSF_ − W_free DSF_)/W_lipid_ × 100 (4)

where W_total DSF_ is the amount of DSF in the formulations, W_free DSF_ is the amount of free DSF determined in the aqueous phase after ultrafiltration–centrifugation (Sartorius^®^ Vivaspin 500 filter unit, 100 kDa molecular weight cut-off), and W_lipid_ corresponds to the weight of the lipid phase of the formulation.

For the determination of W_total DSF_, a certain volume of each formulation was diluted in the mobile phase and sonicated for 15 min at 75 °C. Then, the obtained solution was centrifuged (11,740× *g*) at 4 °C for 5 min and filtered through a 0.2 µm PTFE membrane prior to HPLC analysis. As for the determination of W_free DSF_, NLCs were diluted with cold acetonitrile (in order to guarantee an absence of adsorbed DSF onto the NLCs, as well as drug crystals in the aqueous phase) and ultrafiltrated, with the aqueous phase being analyzed by HPLC. All determinations were performed in triplicate.

#### 2.2.6. Multivariate and Statistical Analyses

The combination of multivariate analysis tools to understand the influence of materials on the properties of nanoparticles (in particular, particle size, polydispersity index, zeta potential, DSF loading, and entrapment efficiency) supports a faster and more robust formulation development [21,22]. Hierarchical clustering (HCA) and principal component (PCA) analyses were used to evaluate the relative similarities between the different formulations. In HCA, Euclidean distances and Ward’s minimum variance method were implemented to determine the distances between clusters, while in PCA, correlations were estimated using the Row-wise method. Here, each principal component derives from an eigenvalue decomposition of the correlation matrix. In parallel, partial least squares (PLS) regression models were implemented with the nonlinear iterative partial least squares (NIPALS) algorithm [23] and validated by leave-one-out cross-validation. Multivariate analyses were conducted on JMP Pro 16 (SAS Institute, Cary, NC, USA), with data being standardized by subtracting the mean and dividing by standard deviation.

Two-way ANOVA (α = 0.05) with Dunnett’s multiple comparison tests were conducted on Graphpad PRISM 8.3.0 (GraphPad Software, San Diego, CA, USA) to assess the statistical significance of differences between groups (*p*-value < 0.05).

## 3. Results and Discussion

### 3.1. Lipid Screening

As stated before, SLNs and NLCs generally consist of three essential components: one or more lipids stabilized by one or more surfactants, dispersed in water. Furthermore, there is no strict rule for the selection of specific lipids for specific drugs. Hence, pre-formulation studies must be conducted to guarantee a suitable excipient selection.

Before selecting potential lipid excipients for formulation development, their immiscibility with water and the achievement of a clear aqueous phase should be warranted, as a (partial) partition of the liquid/solid lipid to the aqueous phase may lead to stability concerns and compromise drug loading. As such, Geloil SC, Labrafil M 2125 CS, Labrafil 1944 CS, Labrasol, Labrasol ALF, Transcutol HP (liquid excipients) and Apifil, Softisan 601, and Suppocire CS2X (solid excipients) were excluded from further analyses.

#### 3.1.1. Liquid Lipid Selection

The incorporation of a liquid lipid into the solid matrix creates a less ordered structure, reducing crystallinity. In addition, drugs are usually more soluble in liquid lipids rather than in solid ones [24]. Medium-chain triglycerides (C_6_ to C_10_) and mixtures thereof, such as Labrafac Lipophile WL 1349 and Miglyol 812 N, are frequently used for NLC development. Synthetic hydroxyl-modified lipids, such as propylene glycol mono- and/or diesters of fatty acids (Capryol PGMC, Capryol 90, Labrafac PG and Lauroglycol 90) may increase the thermodynamic stability of NLCs due to their improved miscibility with solid lipids. Natural pure compounds such as oleic acid, squalene, and squalane are also typically used as NLC excipients. Table 1 introduces commonly employed liquid lipids used for DSF screening.

**Figure 2:**
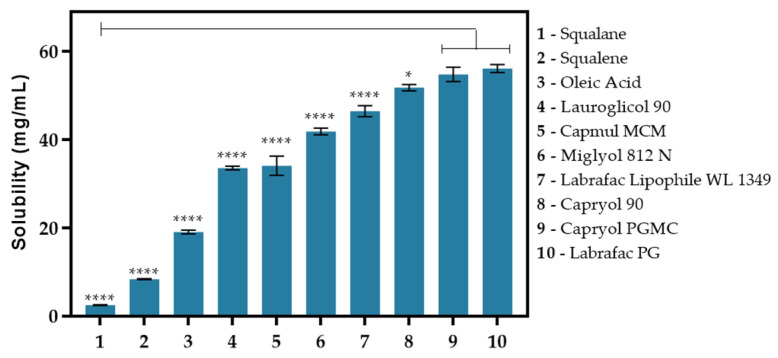
Disulfiram solubility in liquid lipids. * *p*-value < 0.05, **** *p*-value < 0.0001.

The selection of the liquid lipid is of utmost importance, as it conditions the entrapment efficiency and loading of the drug and avoids drug leakage [7,24]. DSF in lipid solubilities (Figure 2) were determined by HPLC. A good solubility of DSF is observed in Labrafac PG (56 ± 1 mg/mL) and Capryol PGMC (55 ± 1 mg/mL), prompting the use of these lipids as potential formulation excipients. On the contrary, natural oils do not show a good ability to solubilize DSF: squalane (2.5 ± 0.1 mg/mL), squalene (8.3 ± 0.1 mg/mL), and oleic acid (19.0 ± 0.4 mg/mL). Notwithstanding, the unsaturated bonds of squalene increase the solubility of DSF when compared to the saturated analogue squalane.

#### 3.1.2. Solid Lipid Selection

There are several solid lipids that can form the solid core of SLNs and NLCs (Table 2), including (i) triglycerides (glycerol esters of three linear saturated fatty acids), (ii) mono and diglycerides (glycerol esters of one or two linear saturated fatty acids and two or one hydroxyl groups), (iii) waxes (esters of fatty alcohols and acids), (iv) fatty acids (saturated linear carboxylic acids (≥C_12_)), and (v) fatty alcohols (saturated linear primary alcohols (≥C_12_)), or mixtures thereof. Their melting point typically ranges from 37 to 80 °C.

**Figure 3:**
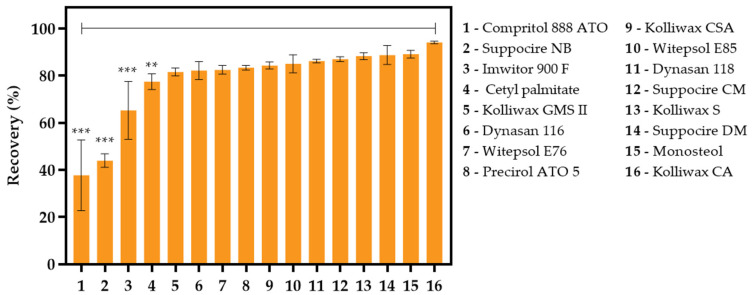
Disulfiram partitioning in binary mixtures of solid lipids and water, determined as recovery (%). ** *p*-value < 0.01, *** *p*-value < 0.001.

Drug partitioning studies were conducted to infer on the lipids that better dissolve or entrap DSF. Increased recoveries indicate that the lipid can accommodate DSF in a higher extent, as opposed to low recovery values, where DSF partitions to the aqueous environment. According to Figure 3, DSF presents a low affinity for Compritol 888 ATO (37 ± 12%), Suppocire NB (44 ± 3%), and Imwitor 900 F (65 ± 10%). On the contrary, there are several lipids that may integrate the lipid core. The top five are Kolliwax CA (94.1 ± 0.4%), Monosteol (89 ± 1%), Suppocire DM (89 ± 3%), Kolliwax S (88 ± 1%), and Suppocire CM (87 ± 1%). Note that all these solid lipids substantially differ in their structure, as well as in their melting points.

#### 3.1.3. Lipid Compatibility and Ratio Selection

Using pure solid lipids for composing the lipid core of SLNs is rarely a good approach, as polymorphic phase transitions during storage can lead to drug leakage [5,6]. Selecting blends of solid lipids or integrating liquid lipids (thus forming NLCs) tends to reduce the matrix crystallinity and improves colloidal stability. Micro- and macroscopic visualization of solid and liquid lipid mixtures is an easy approach for assessing physical compatibility. As a requirement for NLC production, these must display a solid state upon cooling. Phase separation (e.g., Monosteol:Capryol PGMC 1:1), phase heterogeneity (e.g., Kolliwax CA:Capryol PGMC 1:1), and oil adsorption to a paper filter (e.g., Suppocire CM:Labrafac PG 1:1) are signs of incompatibility between lipids (Table 3).

**Figure 4:**
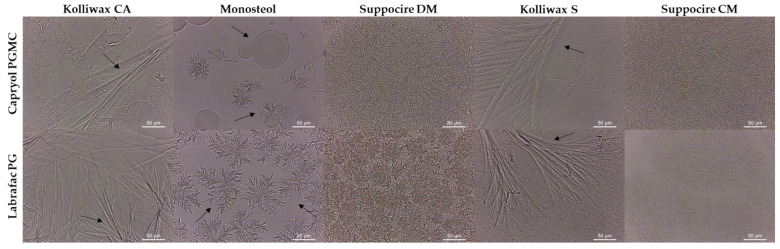
Microscopic evaluation of the physical compatibility of liquid and solid lipid mixtures. Black arrows denote sites of physical instability. Scale bar: 50 µm.

These results are consubstantiated by optical microscopy images (Figure 4). In the samples containing Kolliwax CA (fatty alcohol) and Kolliwax S (fatty acid), the substantial presence of lipid crystals on cooling may indicate possible premature drug expulsion during storage. For instance, Kolliwax S (stearic acid) has four polymorphs (A triclinic and B, C and E monoclinic), as well as various protypes. Upon recrystallization, stearic acid displays the polymorphic form C, which is the most stable at temperatures over 32 °C. However, at lower temperatures and over time, it converts into the highly ordered B form, with drug expulsion [25]. In the ones containing Monosteol, segregated droplets of Capryol PGMC and smaller sized droplets of Labrafac PG near the surface of the solid nuclei are easily observed. As for the samples containing Suppocire CM and Suppocire DM, a more homogeneous phase is seen, which may be ascribed to the composition of these lipids (mixture of mono-, di- and triglyceride esters).

#### 3.1.4. Surfactant Screening

Surfactants, amphiphilic molecules generally consisting of a polar head and one or two hydrophobic tails, are crucial for the long-term stability of nanoparticles. As SLNs/NLCs are somehow similar to lipid nano- and microemulsions, they adapt the same concepts for stabilization of oil-in-water emulsions: the surfactant tails should be positioned near the hydrocarbon tails of the lipid in the interface, with their polar heads facing the external aqueous phase. The choice of surfactants depends mainly on the lipid matrix, since they need to be compatible, and it is critical for obtaining NLCs with the desired colloidal properties, particularly, particle size, distribution, and zeta potential. The vast majority of surfactants employed for NLC development are non-ionic and are listed in Table 4.

For screening purposes, NLCs composed of Suppocire DM:Capryol PGMC (1:1), Suppocire DM:Labrafac PG (1:1), and Suppocire CM:Capryol PGMC (1:1), with 5% of an *o/w* surfactant and 1% of a *w*/*o* co-surfactant were systematically produced by high-pressure homogenization. Process parameters were fixed, in order not to add additional variability sources. The stabilization of lipid emulsions may benefit from using surfactants with equivalent hydrocarbon chains, hence the use of the pairs Tween 20/Span 20, Tween 40/Span 40, Tween 60/Span 60, and Tween 80/Span 80 [26,27].

**Figure 5:**
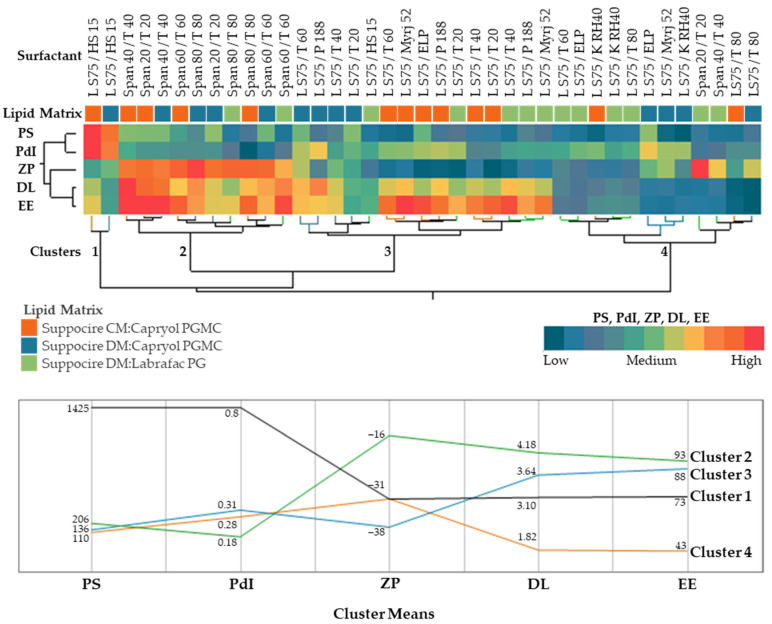
Hierarchical clustering analysis showing similarities in terms of colloidal properties (Particle Size, PS, Polydispersity Index, PdI, and Zeta Potential, ZP), disulfiram loading, DL, and entrapment efficiency, EE.

The hierarchical clustering of the formulations, evaluated 15 days after production, separates them in four major clusters: Cluster 1, with increased particle size and dispersion, Cluster 2, with the lowest |zeta potential| and the highest DSF loading, and Clusters 3 and 4, differing mainly on DSF loading and entrapment efficiency (Figure 5). Cluster 1 encompasses drug delivery systems with pronounced instability, with loss of colloidal size and increased particle aggregation (day 1, PS = 42 ± 2 nm, PdI = 0.21, ZP = −34 ± 1 mV vs. day 15, PS = 1609 ± 97 nm, PdI = 0.914, ZP = −33 ± 1 mV). Both formulations were emulsified with Kolliphor HS 15. ZP values higher than |30| mV are reported to predict good stability, as it allows a sufficient electrostatic repulsion between dispersed nanoparticles [28]. The production of carriers complying to this thumb rule but lacking in stability is common, indicating that ZP alone may not be a good predictor of stability. In fact, surfactants and other surface modifications (e.g., polyethylene glycol, chitosan) may also provide additional steric stabilization, in which their polymeric structure reduces particle interactions, despite the lower magnitude of ZP values [29]. Cluster 2 groups the majority of formulations constituted by the Span X/Tween X combinations, which are characterized by a nanometric size and monomodal distribution as well as favorable entrapment efficiencies and drug loading. Cluster 3 includes several NLCs with reduced size but somehow polydisperse and stabilized by several surfactants, including Lipoid S75/Tween 20, Lipoid S75/Tween 40, and Lipoid S75/Tween 60. Cluster 4 includes unfavorable NLCs in terms of drug encapsulation, despite their reduced size. Interestingly, NLCs produced with Lipoid S75/Tween 80 belong to this cluster.

**Figure 6:**
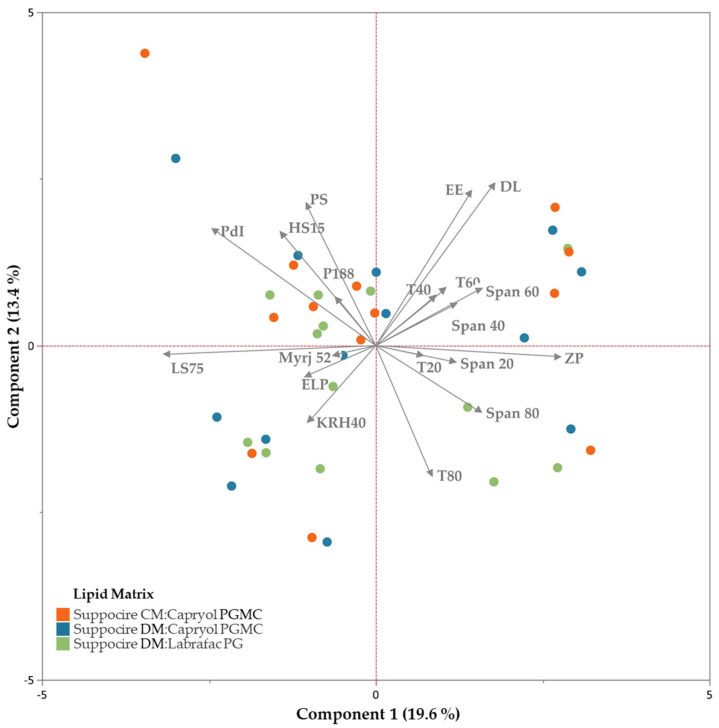
Biplot representation of NLCs and the corresponding composition variables, on the first two principal components, recovering 33% of variance.

The composition trends of NLCs can be evaluated through the representation of the first two principal components in a Cartesian plane, in which the association between NLC excipients and colloidal properties are discriminated (Figure 6). Tweens/Spans and other surfactants play a different role in colloidal properties, as they are distributed in different quadrants (right and left, respectively). In fact, the first principal component distributes formulations based on their surfactants. Accordingly, polysorbates in combination with the respective alkyl sorbitan esters are efficient emulsifiers for a matrix composed by Suppocire CM:Capryol PGMC, which also maximize DSF loading and entrapment while maintaining a monomodal size distribution. Nonetheless, their emulsification efficiency is similar and do not show significant differences between themselves. Kolliphor RH40, Kolliphor ELP, Kolliphor P188, and Myrj 52 also show a similar behavior in stabilizing the formulations.

**Figure 7:**
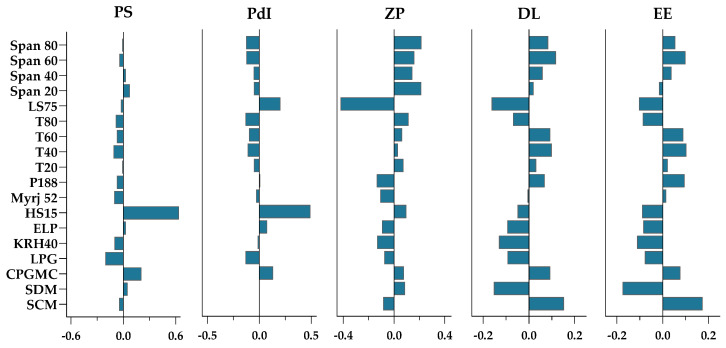
Partial least squares regression coefficients for surfactants, liquid, and solid lipids, considering five responses: particle size (PS), polydispersity index (PdI), zeta potential (ZP), drug loading (DL), and entrapment efficiency (EE) of disulfiram encapsulating NLCs.

In PLS regressions, the variables with the highest magnitude show a direct (positive sign) or indirect (negative sign) correlation with the respective response. Further insights into the impact of excipients on the NLC properties is given in what follows. According to Figure 7, the incorporation of Labrafac PG into the lipid matrix reduces particle size and polydispersity in contrast to the use of Capryol PGMC. This is in accordance with the viscosity of the oils (Table 1), as an increased viscosity of the lipid phase tends to form bigger nanoparticles due to the less efficient formation of emulsified oil droplets. However, NLCs containing Suppocire DM:Labrafac PG showed a lower drug loading and entrapment efficiency, ruling out these lipids as favorable excipients for DSF delivery. As also evidenced by HCA and PCA, using Kolliphor HS 15 led to particle aggregation with consequent polydispersity and drug expulsion. The use of SPAN X as a co-surfactant significantly increases zeta potential, contrasting with Lipoid S75, with Span 60/Tween 60 standing out as the best surfactants for maximizing drug loading and encapsulation. Furthermore, the use of Suppocire CM:Capryol PGMC as the constituents of the lipid matrix not only favors drug loading and entrapment efficiency but also leads to smaller and monodisperse NLCs.

## 4. Conclusions

The application of solid lipid nanoparticles and nanostructured lipid carriers for drug delivery is virtually unlimited. Such systems have demonstrated robust preclinical efficacy and safety, particularly in tumor therapy. Although lipid nanoparticles can be developed for any form of cancer, the selection of excipients must take into account the route of administration.

The wider range of potential excipients is a double-edged sword: lipid nanoparticles are extremely diverse in composition, which increases the potential for high drug loading and stability. On the other hand, the structural complexity of lipids and the availability of surfactants, as well as the interactions and compatibility of materials, often makes carrier optimization difficult to predict. The choice of lipid is determined by the solubility of the drug in the lipid, with higher solubility naturally favoring the partition and entrapment of lipophilic drugs. In addition, the incorporation of a liquid lipid into the solid matrix leads to the formation of NLCs. Liquid and solid lipids should not be formulated into NLCs without a prior evaluation of the physicochemical compatibility of the matrix. Subsequently, a surfactant, or a mixture thereof, should be able to effectively emulsify the colloidal dispersion and reduce its size to the nanometer range while providing electrostatic or steric stability.

Here, using disulfiram as a model anticancer drug, NLCs were prepared by sequential design and excipient carrier interaction evaluation, either by analytical, optical, or multivariate analysis tools. Using a lipid matrix of Suppocire CM:Capryol PGMC stabilized by Span 60/Tween 60, monodisperse NLCs, with small nanometric size, monodisperse and optimized loading and entrapment efficiency were developed. Overall, the comprehensive step-by-step workflow studied here encompasses the successive stages of nanoparticle preparation and provides a straightforward to simple but effective methodology that can help researchers develop suitable lipid nanoparticles.

## Figures and Tables

**Table 1:** List of frequently used liquid lipids (oils) as excipients for NLC development *.

Trademark Name	EP Name/USP Name	Chemical Description	Viscosity (mPa·s, 20 °C)	HLB
Capmul MCM	-/Medium-chain mono- and diglycerides	Medium-chain mono- and diglycerides of caprylic (C_8_) and capric (C_10_) acids	-	6
Capryol PGMC	-/Propylene glycol monocaprylate (type I)	Propylene glycol esters of caprylic acid (C_8_), composed of mono- and diesters	20	6
Capryol 90	-/Propylene glycol monocaprylate (type II)	Propylene glycol esters of caprylic acid (C_8_), mainly composed of monoesters and a small fraction of diesters	20	5
Labrafac Lipophile WL 1349	Triglycerides, medium-chain/Medium-chain triglycerides	Medium-chain triglycerides of caprylic (C_8_) and capric (C_10_) acids	25–33	1
Labrafac PG	Propylene glycol dicaprylocaprate/Propylene glycol dicaprolate/dicaprate	Propylene glycol esters of caprylic (C_8_) and capric (C_10_) acids	9–12	1
Lauroglycol 90	Propylene glycol monolaurate (type II)/Propylene glycol monolaurate (type II)	Propylene glycol mono- and di- esters of lauric (C_12_) acid, mainly composed of monoesters and a small fraction of diesters	25	3
Miglyol 812 N	Triglycerides, medium-chain/Medium-chain triglycerides	Medium-chain triglycerides of caprylic (C_8_) and capric (C_10_) acids	30	-
Oleic Acid	Oleic Acid/*Cis*-9-octadecenoic acid	Monounsaturated omega-9 octadecenoic (C_18_) fatty acid	40	1
Squalane	Squalane/Hydrogenated C_30_ hydrocarbon	Hydrogenated C_30_ isoprenoid hydrocarbon	31	11
Squalene	Squalene/C_30_ isoprenoid hydrocarbon	C_30_ isoprenoid hydrocarbon	12	-

* information provided by the manufacturers.

**Table 2:** List of frequently used solid lipids as excipients for SLN and NLC development *.

Trademark Name	EP Name/USP Name	Chemical Description	Melting Point (°C)	HLB
Cetyl palmitate	Cetyl palmitate/Cetyl palmitate	Hexadecyl hexadecanoate, the ester derived from hexadecanoic acid and hexadecanol	54–55	10
Compritol 888 ATO	Glycerol dibehenate/Glyceryl dibehenate	Mono-, di-, and triesters of behenic acid (C_22_), the diester fraction being predominant	65–77	2
Dynasan 116	-/Tripalmitin	Glyceryl triester of palmitic (C_16_) acid	64	-
Dynasan 118	-/Glyceryl Tristearate	Glyceryl triester of stearic (C_16_) acid	72	-
Geleol FPF	Glycerol Monostearate 40–55 (Type I)/Mono and Diglycerides	Mono-, di-, and triesters of palmitic (C_16_) and stearic (C_18_) acids, the mono fraction being predominant	56–64	3
Geleol mono/diglycerides NF	Glycerol Monostearate 40–55 (Type I)/Mono and Diglycerides	Mono-, di-, and triesters of palmitic (C_16_) and stearic (C_18_) acids, the mono fraction being predominant	54–64	3
Imwitor 900 F	Glycerol Monostearate 40–55 (Type I)/Mono and Diglycerides	Mono-, di-, and triesters of palmitic (C_16_) and stearic (C_18_) acids, the mono fraction being predominant	59	3
Kolliwax CA	Cetyl Alcohol/Cetyl Alcohol	Hexadecan-1-ol, C_16_ alcohol	49–50	-
Kolliphor CSA	Cetostearyl Alcohol (Type A), Emulsifying/Cetostearyl Alcohol	Mixture of cetyl (C_16_) and stearyl (C_18_) fatty alcohols with the anionic emulsifier sodium cetostearyl sulphate	48–56	7
Kolliwax GMS II	Glycerol Monostearate 40–55 (type II)/Mono and Diglycerides	Mono-, di-, and triesters of palmitic (C_16_) and stearic (C_18_) acids, the mono fraction being predominant	54–64	3.8
Kolliwax S	Stearic Acid/Stearic Acid	1-heptadecanecarboxylic acid, C_18_ acid	69–70	-
Monosteol	Propylene glycol monopalmitostearate/-	Propylene glycol esters of palmitic (C_16_) and stearic (C_18_) acids, the monoester fraction being predominant	33–40	4
Precirol ATO 5	Glycerol distearate (type I)/Glyceryl distearate	Esters of palmitic (C_16_) and stearic (C_18_) acids, the diester fraction being predominant	50–60	2
Suppocire CM	Hard fat/Hard fat	Mono-, di-, and triglyceride esters of fatty acids (C10 to C_18_), the triester fraction being predominant	36–40	-
Suppocire DM	Hard fat/Hard fat	Mono-, di-, and triglyceride esters of fatty acids (C_10_ to C_18_), the triester fraction being predominant	42–45	-
Suppocire NB	Hard fat/Hard fat	Mono-, di-, and triglyceride esters of fatty acids (C_10_ to C_18_), the triester fraction being predominant	35–39	-
Witepsol E76	Hard fat/Hard fat	Hydrogenated coconut mono-, di-, and triglycerides	37–39	-
Witepsol E85	Hard fat/Hard fat	Hydrogenated coconut mono-, di-, and triglycerides	42–44	-

* information provided by the manufacturers.

**Table 3:** Physical compatibility of solid and liquid lipid mixtures.

		Liquid Lipids				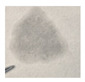
		Capryol PGMC		Labrafac PG		
		Macro	Micro	Macro	Micro	
**Solid Lipids**	Kolliwax CA	X *	X *	X *	X *	+ Control
	Kolliwax S	X *	X *	X *	X *	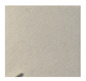 − Control
	Monosteol	X	X	X	X	
	Suppocire CM	Y	Y	X	Y	
	Suppocire DM	Y	Y	Y	Y	

* Crystallization of the solid lipid. The positive control represents a sample with macroscopic physical incompatibility between the liquid and the solid lipid, as shown by lipid adsorption on the filter. The negative control represents a sample with macroscopic physical compatibility between lipids and lack of oil adsorption on the filter paper.

**Table 4:** List of commonly used non-ionic surfactants as excipients for NLC development *.

Trademark Name	EP Name/USP Name	Chemical Description	HLB	IV Approved
Kolliphor ELP	Polyoxyl castor oil/Polyoxyl 35 castor oil	Purified polyethoxylated castor oil	12–14	Yes
Kolliphor HS 15	Macrogol 15 hydroxystearate/-	Polyglycol mono- and di-esters of 12-hydroxystearic acid with 30% of free polyethylene glycol	14–16	Yes
Kolliphor P 188	Poloxamers/Poloxamer	Poloxamer 188, block copolymer of propylene oxide and ethylene oxide (79.9–83.7%)	>24	Yes
Kolliphor RH40	Macrogolglycerol hydroxystearate/Polyoxyl 40 hydrogenated castor oil	Polyoxyl 40 hydrogenated castor oil	14–16	No
Myrj 52	Macrogol stearate/Polyoxyl 40 stearate	Polyoxyethylene (40) monostearate	17	No
Tween 20	Polysorbate 20/Polysorbate 20	Partial esters of lauric acid with sorbitol and its anyhydrides ethoxylated with ethylene oxide (1:20)	16.7	Yes
Tween 40	Polysorbate 40/Polysorbate 40	Partial esters of palmitic acid with sorbitol and its anyhydrides ethoxylated with ethylene oxide (1:20)	15.6	No
Tween 60	Polysorbate 60/Polysorbate 60	Partial esters of stearic acid (50) with sorbitol and its anyhydrides ethoxylated with ethylene oxide (1:20)	14.9	No
Tween 80	Polysorbate 80/Polysorbate 80	Partial esters of oleic acid with sorbitol and its anyhydrides ethoxylated with ethylene oxide (1:20)	15	Yes
Lipoid S75	-/-	Fat-free soybean phospholipids with 70% phosphatidylcholine, 7.5% phosphatidylethanolamine and 2.5% lysophosphatidylcholine	8–11	Yes
Span 20	Sorbitan laurate/Sorbitan monolaurate	Esters of sorbitol and its mono- and di-anhydrides with lauric acid	8.6	No
Span 40	Sorbitan palmitate/Sorbitan monopalmitate	Esters of sorbitol and its mono- and di-anhydrides with palmitic acid	6.7	No
Span 60	Sorbitan stearate/Sorbitan monostearate	Esters of sorbitol and its mono- and di-anhydrides with stearic acid 50	4.7	No
Span 80	Sorbitan oleate/Sorbitan monooleate	Esters of sorbitol and its mono- and di-anhydrides with oleic acid	4.3	No

* information provided by the manufacturers/FDA ‘Inactive Ingredients Database’, version of 10 October 2021.

## Data Availability

The raw data required to reproduce these findings are available on reasonable request from the corresponding author (C.V.).
